# Progress of near-infrared-II fluorescence in precision diagnosis and treatment of colorectal cancer

**DOI:** 10.1016/j.heliyon.2023.e23209

**Published:** 2023-12-03

**Authors:** Yong Wu, Hongtao Cao, Shaoqing Yang, Chaohui Liu, Zhenguo Han

**Affiliations:** Third Hospital of Shanxi Medical University, Shanxi Bethune Hospital, Shanxi Academy of Medical Sciences, Tongji Shanxi Hospital, Taiyuan, 030032, China

**Keywords:** NIR-II, colorectal cancer, precision diagnosis and treatment

## Abstract

Colorectal cancer is a malignant tumour with high incidence and mortality worldwide; therefore, improving the early diagnosis of colorectal cancer and implementing a targeted "individualized treatment" strategy is of great concern. NIR-II fluorescence imaging is a large-depth, high-resolution optical bioimaging tool. Around the NIR-II window, researchers have developed a variety of luminescent probes, imaging systems, and treatment methods with colorectal cancer targeting capabilities, which can be visualized and image-guided in clinical surgery. This article aims to overcome the difficulties in diagnosing and treating colorectal cancer. The present review summarizes the latest results on using NIR-II fluorescence for targeted colorectal cancer imaging, expounds on the application prospects of NIR-II optical imaging for colorectal cancer, and discusses the imaging-guided multifunctional diagnosis and treatment platforms.

## 1 Introduction

Colorectal cancer is one of the common malignant tumours of the digestive system, with extremely high morbidity and mortality [1]. The low rate of early diagnosis and the high rate of recurrence and metastasis are still the bottlenecks that hinder the long-term survival of colorectal cancer patients. Currently, the treatment methods for colorectal cancer mainly include traditional surgery, radiotherapy, chemotherapy and immunotherapy Table 1. However, colorectal cancer is a highly heterogeneous disease that can cause irreparable harm to patients if not treated properly. Therefore, a precise treatment plan will enable patients to receive the most appropriate treatment and avoid unnecessary over-treatment to improve their quality of life [2]. With molecular imaging and endoscopic techniques for early diagnosis and borderline precision of colorectal cancer in clinical practice, each diagnostic modality possesses unique advantages but also has many shortcomings [3,4]. According to the individual characteristics of the disease, the effective integration of various molecular imaging techniques to comprehensively obtain information on the lesion site has unparalleled advantages in accurate diagnosis [3,5].

**Table 1:** Benefits and limitations of different therapy methods.

Therapy Method	Benefits	Range	Limitations
Surgery	Surgery is the mainstay curative treatment for patients with non-metastasized colorectal cancer.	Local	Limited efficacy in the treatment of advanced tumours; alters to some extent the quality of patients' postoperative survival.
Chemotherapy	Combination chemotherapy is the mainstay treatment for metastatic colorectal cancer; it reduces the recurrence of the tumour to a certain extent.	Systemic	Drug resistance and toxic effects of chemotherapeutic agents.
Radiotherapy	Short-term reduction of local tumor volume.	Local	More adverse reactions.
Immunotherapy	Selective enhancement of the host immune response to the tumour.	Systemic	Only target at microsites lite-instability high/mismatch repair-deficient metastatic colorectal cancers.
Targeted therapy	Precision treatment; There are relatively few adverse reactions.	Systemic	There are few effective therapeutic targets for colorectal cancer; only for some colorectal cancer patients.

The luminescent biological tissue in the second near-infrared (NIR-II, 1000–1700 nm) owing to reduced photon scattering, absorption and lower tissue autofluorescence， leading to less image distortion and higher spatial resolution, and allows fluorescence imaging to show deeper penetration in biological tissues [6]. From the above advantages, NIR-II fluorescence imaging has better light penetration, lower background signal, higher safety limits, and higher Maximum Permissible Exposure (MPX). It has the advantages of easy operation, sensitivity, non-invasive, non-radioactive and so on. It can directly collect real-time dynamic biological events in vivo to achieve an accurate diagnosis and intraoperative guidance [7,8]. By combining various technologies, therapeutics promise to provide effective targeted therapy and improve treatment outcomes, showing great promise in diagnosing disease, monitoring disease progression, guiding surgery, and evaluating treatment effects [3,9,10].

The use of NIR for accurate diagnosis and treatment of colorectal cancer will become the most potential new direction for the treatment of colorectal cancer. In this review, we focus on the recent progress of NIR-II fluorescence probes with colorectal cancer targeting ability. We further discuss the recent progress in colorectal cancer imaging and therapy based on NIR-II imaging.

### 1.1 NIR-II fluorescence probes with characteristics targeting colorectal cancer

**Fig. 1:**
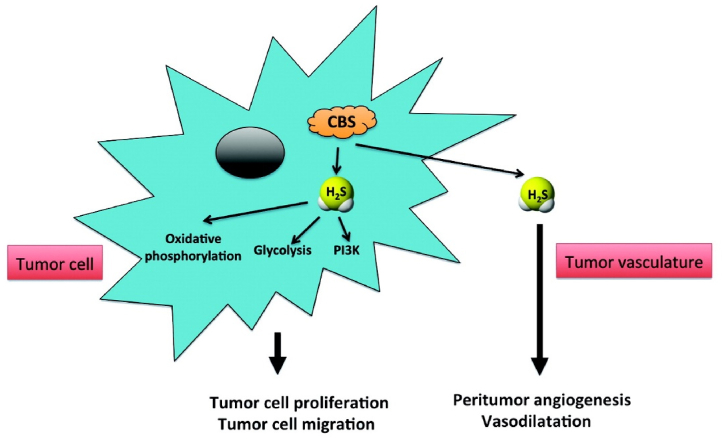
Stimulation of colorectal cancer cell growth, proliferation, migration, and peritumor angiogenesis and blood flow by the CBS/H2S axis.. (Reproduced with permission from Csaba Szabo & Mark R Hellmich [17]. Copyright 2013 Cell Cycle.)

Like nitrogen oxide (NO) and carbon monoxide (CO), hydrogen sulphide (H_2_S) is one of the gassignalling molecules in the body. It plays a very critical role in many pathophysiological processes in the body. Studies have found that H2S is believed to be associated with the occurrence and development of tumours [11]. In intestinal tissue, H2S is mainly produced by cystathionine-β-synthase (CBS) [12]. The researchers observed that CBS was selectively upregulated in colorectal cancer tissues compared to normal mucosal tissues. In addition, sulfide detoxifying enzymes are reduced in colorectal cancer tissues [[13], [14], [15], [16], [17], [18], [19], [20], [21]]. Utilizing the property of increased concentration of H2S at the lesion site could be a potential therapeutic target for the treatment and diagnosis of cancer. Fig. 1.

**Fig. 2:**
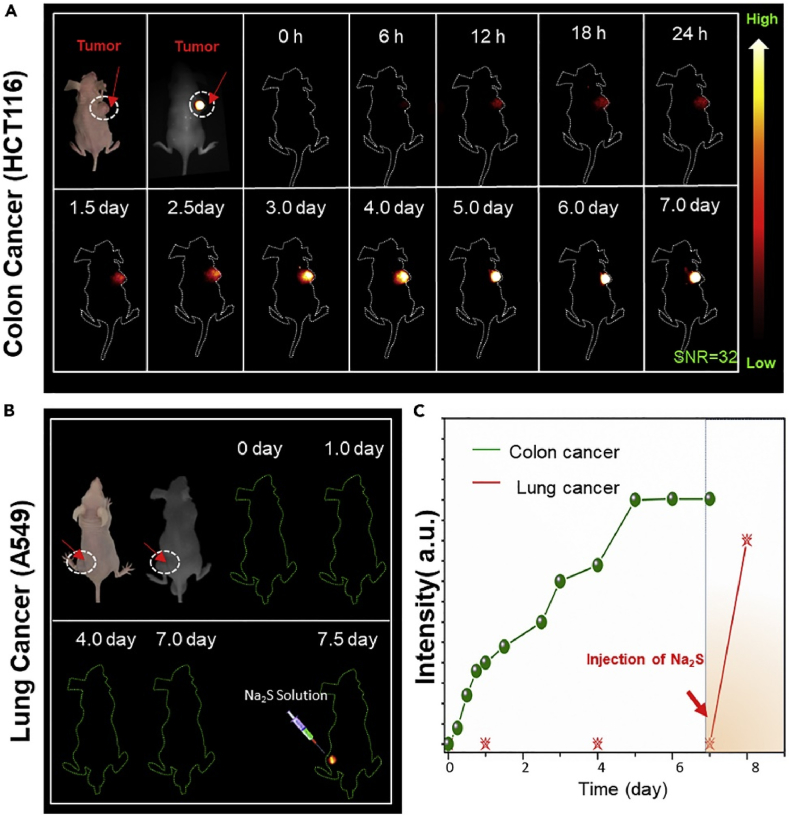
(A–C) In Vivo-Specific Visualization of Colorectal Cancer Based on In Situ Endogenous H2S Activation of Ag-CEW Complex. (A) Colon (HCT116) tumor-bearing mouse. (B) Lung (A549) tumor-bearing mouse. (C) The mean NIR-II signal intensity change in the tumor region from 1 to 7 days. (Reproduced with permission from Deng.et al. [25]. Copyright 2019 iScience.).

Due to the peculiar properties of H_2_S, the three main strategies for the H_2_S reaction probe are reduction of the nitride to amine, nucleop hilic reaction, and S-precipitation [22]. Specific fluorescent dyes enable fluorescence emission after these reactions with H_2_S. The intensity of their fluorescence depends on the concentration of H_2_S units. The higher the engagement, the higher the degree of response and the better the targeting ability of the tumour. Although there is a wide variety of fluorescent probes based on H_2_S reactions, their simple synthesis, non-toxicity and fast metabolism are essential for practical applications. Recent reports on three nano-fluorescence platforms using H_2_S-induced in situ reduction reactions to form Ag_2_S quantum dots to target colorectal cancer [[23], [24], [25]] Fig. 2(A-C), enabling tumour localization of NIR-II fluorescence, photothermal therapy (PTT), and targeted release of chemotherapeutics. Using the Nucleophilic Aromatic Substitution reaction (SNAr) of H_2_S, Xu et al. selectively identified H_2_S-rich colorectal cancer cells by the specific and proportional fluorescence response exhibited by two organic fluorophores to H_2_S and distinguished live cell types based on their differences in H_2_S content in two-colour imaging mode [10]. Tian et al. utilized colorectal cancer to produce high concentrations of H_2_S, accelerated Fe(III)/Fe(II) conversion, and photothermal synergistically enhanced reactive oxygen species (ROS) production, thereby inhibiting tumour cell growth [26].

In addition, several upregulated receptors exist on colorectal cancer cell membranes, including antibodies, lectins, small peptides and small targeting molecules [[27], [28], [29], [30]]. Fluorescent stains can actively target tumours for diagnostic and therapeutic purposes by coupling to these molecules that recognize tumour-specific biomarkers. For example, immunotherapy based on immune checkpoint blockade of programmed cell death-1 (PD-1) and its ligand-1 (PD-L1) for colorectal cancer treatment has yielded promising results. The combination of NIR-II fluorescence with PD-L1 provides a more accurate prediction of immunotherapy response and effective tumour diagnosis [31]. Integrin αvβ6 is proving to regulate many malignant behaviours in colorectal tumour cells. Many classical oncogenic signalling pathways are involved in integrin αvβ6-mediated colorectal cancer progression [32]. Cyclic arginine-glycine-aspartate(cRGD) is a peptide that binds to integrins and can be coupled to fluorescent dyes, allowing for more accurate and precise localization of colorectal tumours [[33], [34], [35]].

The proposed concept of NIR-II fluorescence dramatically promoted the development of NIR-II probes, providing powerful tools for precision diagnosis and treatment of colorectal cancer. It is worth noting that although NIR-II fluorescence probes hold excellent properties, they are still in their infancy, and some fundamental issues need to be addressed for their further applications in clinical practice, mainly reflected in the following. (1) False-positive results. It is mainly due to differences in biomarker concentrations in diseased and normal tissues, e.g., CBS highly expresses H2S. At the same time, normal intestinal tissues also produce H2S, which may lead to false-positive results. On the one hand, the probes can be activated in a dual-response manner to increase specificity. On the other hand, the occurrence of false positives can be minimized by setting up the detection line. (2) The low number of existing NIR-II probes with characteristics of targeting colon cancer and the insufficient number of regulatory sites are the main obstacles limiting the exploration of their further applications. Currently, the primary method to improve the targeting ability is to modify the target of the fluorescent probe to improve the specificity of the diseased tissue. Secondly, by improving the drug delivery ability, the fluorescent probe is delivered to the target tissue and its concentration in the target tissue is increased. (3) Safety and biocompatibility. Most NIR-II probes still have some toxic side effects on the human body. Even now, no FDA-approved NIR-II probes are available for clinical use. Therefore, safety studies should be strengthened for clinical applications. Although long-term efforts are needed to address these challenges, NIR-II probes hold promise for widespread use in colorectal cancer. Successful clinical application of NIR-II fluorescent probes will contribute to the accurate and effective diagnosis or treatment of colorectal cancer.

### 1.2 Diagnosis of colorectal tumours with NIR-II fluorescence

Colorectal cancer, as per morphological classification, can be divided into three types: ulcerative, mass and infiltrative. As per histopathology, there are three types: adenosquamous epithelial carcinoma, squamous cell carcinoma and carcinoid carcinoma. It is still challenging to locate and diagnose colorectal cancer accurately. Clinicians prefer computed tomography (CT) [36] and e-colonoscopy [37] to diagnose colorectal tumours. However, only obvious tumours can be analyzed, and colonoscopy often damages the bowel. Table 2.

**Table 2:** Benefits and limitations of colorectal cancer diagnostic techniques.

Benefits	Limitations	M staging	N staging	T staging	
Three-dimensional CT provides a great deal of information regarding vascular anatomy, which can assist in planning laparoscopic resections [[93], [94], [95]].	Radiation exposure; The effect of soft tissue imaging was poor. Imaging of small tumours and metastatic tumours (<3 mm in diameter) is challenging [96].	CT colonography demonstrates liver metastases, pulmonary metastases and other sites of disease. The sensitivity of liver metastases detected by CT is 85 % and the specificity is 97 % [97,98].	Metastatic lymph nodes tend to be more than 1 cm in diameter, and have a circular shape, irregular border, central necrosis, and calcifications. The overall accuracy is 59%–71 % [[98], [99], [100]].	Wall deformities are associated with a specific T stage. The overall accuracy for T stage is 73%–83 % [99,101,102].	CT colonography
Soft tissue imaging is good; chemoradiation therapy evaluation and the identification of liver metastases [98,103].	The imaging time is long, which is easily affected by intestinal peristalsis and contents. Imaging of small tumours and metastatic tumours (<3 mm in diameter) is challenging [96,104].	Enhanced MRI has become a first-line imaging modality to identify liver metastases [105].	Sensitivity 80%–85 % and specificity 97%–98 % by using border irregularity and mixed intensity signal intensity of metastatic lymph nodes [106,107].	High-resolution T2-weighted imaging is the key sequence. Evaluation of the circumferential resection margin is important [98].	MRI
Pathological diagnosis is the gold standard for cancer diagnosis [108].	Invasive procedure; complications occurred such as hemorrhage, perforation	The main local changes of intestinal mucosa in early colorectal cancer were observed by gross morphology. The intestinal wall tissue was taken out for pathological examination to determine whether there was disease [108,109].			e-colonoscopy
It is used to diagnose small primary tumours and metastases less than 3 mm in diameter with high spatial resolution and high temporal resolution. It can be excited single or multiple times, which is convenient for multiple imaging in vivo, and can realize multi-channel imaging with MRI, CT, photoacoustic imaging, etc. [6,50,52,96]	There are no clinically approved fluorescent dyes; poor penetration; no anatomical information.	NIR-Ⅱ can be used for accurate imaging of blood vessels and lymphatic system, and has good imaging effect on small tumours and their microvessels. It has high permeability and long retention effect, and can identify small tumours and metastatic tumours in vivo. It can be used to guide sentinel lymph node surgery in a variety of tumor models, and can also be used to study the process of tumor cell proliferation, invasion and metastasis in real time [[110], [111], [112]].			NIR-II fluorescence imaging

**Fig. 3:**
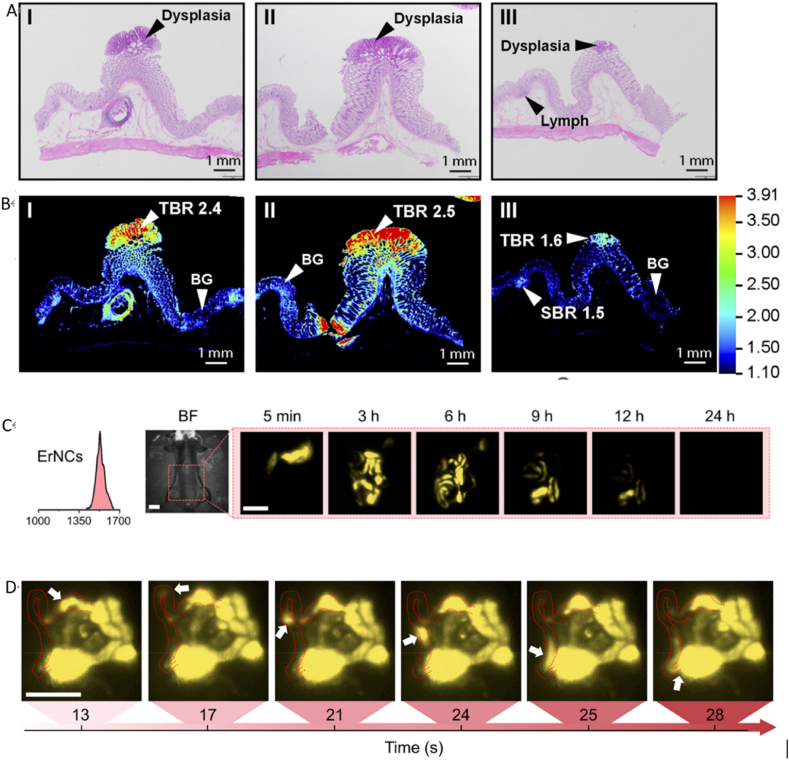
(A–D) (A) (B) Colon tissue slices stained with H&E and their corresponding NIR image scans (Reproduced with permission from Yim, J.J. et al. [37]. Copyright 2020 Biological Sciences.). (C) Time-course NIR-II imaging performed at 5 min, and 3, 6, 9, 12 and 24h post the gavage to show the high spatial resolution achieved. (min: minutes, h: hours, BF: bright field). (D) High temporal resolution of NIR-IIb imaging for real-time monitoring of the dynamics of GI peristalsis and meal flow in the intestinal tract. The white arrows show the peristalsis and meal flow direction. (Reproduced with permission from Mi, C. et al. [49]. Copyright 2022 Nano Letters.).

**Fig. 4:**
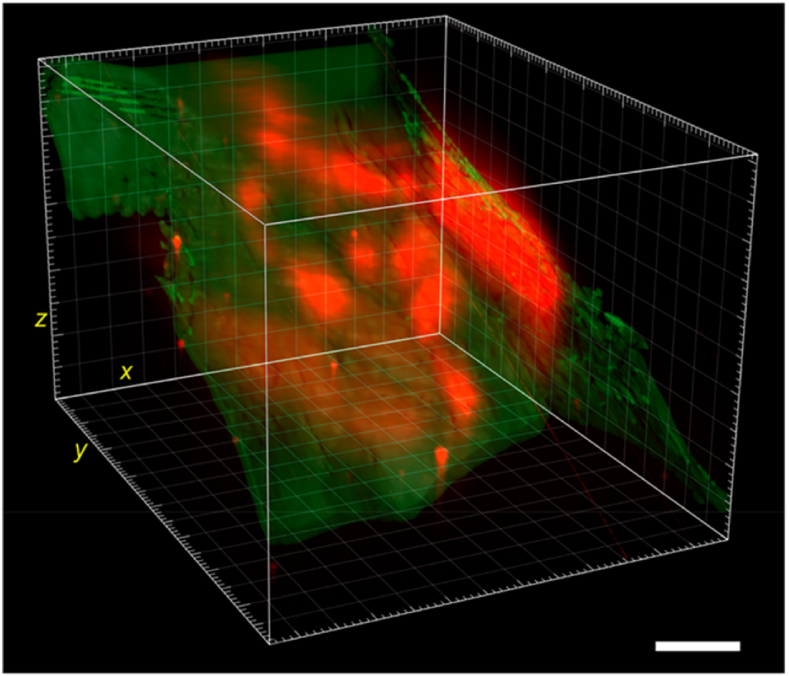
Three-dimensional imaging of GI tract by a light-sheet illumination system. Green: skin. Red: GI tract. (Reproduced with permission from Mi, C. et al. [49]. Copyright 2022 Nano Letters.). (For interpretation of the references to color in this figure legend, the reader is referred to the Web version of this article.)

Due to the advantages of low photon scattering and autofluorescence for deep tissue penetration and high spatial resolution, NIR-II fluorescence imaging offers an alternative tool to non-invasive imaging modalities [[38], [39], [40], [41], [42], [43], [44], [45]].Moreover it does have applications for in vivo visualization of the vascular system, organs, tumours, and the gastrointestinal tract [45,46]. Hong et al. performed non-invasive and real-time NIR-II imaging of the gastrointestinal tract in health and disease to study normal gastrointestinal dynamics and intestinal obstruction in mice [47]. Li et al. demonstrated the NIR-II fluorescence imaging of the GI tract by engineering a protein corona structure consisting of a ribonuclease-A (RNase-A) on the particle surfaces [48]. Chao et al. enhanced the spatial resolution tõ1 mm in GI tract imaging and realized a high temporal resolution of 8 frames per second in NIR-IIb fluorescence imaging. Furthermore, it demonstrated a three-dimensional (3D) imaging of the GI tract. In addition, they succeeded in diagnosing intestinal diseases through 3D imaging of the gastrointestinal tract [49]. Fig. 3(A-D) and Fig. 4.

When applied as a diagnostic method, colorectal-targeted probes allow clear visualization of small changes in the colorectal, providing a valuable source of information for early diagnosis. In addition, the indistinguishable borders of colorectal tumours are forcing surgeons to balance the need to reduce the rate of positive margin cuts with the need to maintain bowel function. Metastasis from colorectal cancer is a critical point that leads to difficulty in treatment and poor prognosis. Several pathological processes occur during the development of metastasis from colorectal cancer. These processes include tumour cell invasion and angiogenesis. Early detection and localization of small colorectal tumour metastases can prevent the further spread of tumour cells. High-quality display of in vivo biological information at large depths thanks to the high-performance NIR-II light-emitting probe, we assume that future screening of colorectal tumours will rely on NIR imaging and provide accurate tumour margin information preoperatively and intraoperatively.

However, in clinical applications, a single imaging mode is not available to effectively provide comprehensive information about tumour morphology and function, and single-modality imaging is often unable to perform a complete and accurate assessment of colorectal cancer. The development of colorectal cancer is the result of multiple pathways, multiple processes and multiple stages of a complex process. Thus, combining two or more different diagnostic modalities is a promising approach, which may lead to synergistic effects. Hu et al. constructed a three-modality imaging platform of NIR-II, Photoacoustic Imaging (PAI), and Magnetic Resonance Imaging (MRI) to achieve multimodal diagnosis and treatment of cancer [50]. Guided by constructing an integrated diagnostic and therapeutic platform of MRI and PAI, Zhang et al. used nanoprobe-induced chemotherapy and hyperthermia to treat colorectal cancer mice in vivo [51]. Simultaneously, Dai et al. achieved tumour precision imaging using NIR-II optical imaging with CT imaging [52]. The development of these multimodal composite imaging techniques is promising to show great promise for diagnosing and treating colorectal cancer [3]. Table 3.

**Table 3:** Multimodal imaging.

Nanoplatform/Nanoprobes	Mode of imaging	Treatment mode
PFTQ-PEG-Gd NPs [50]	NIR-II fluorescence imaging MRI photoacoustic imaging	PTT
AMG@CM [113]	NIR-II fluorescence imaging MRI photoacoustic imaging photothermal imaging	PTT chemodynamic therapy
NaYF4: Nd/NaDyF4 nanocrystals [114]	NIR-II fluorescence imaging MRI	
Au Nanosheets and GdOF:Yb,Er [115]	NIR-II fluorescence imaging MRI CT	PTT PDT
QT-RGD [116]	NIR-II fluorescence imaging photoacoustic imaging CT	PTT
TPATQ-PNP NPs [117]	NIR-II fluorescence imaging MRI	PTT
Rare-Earth-Metal (Nd3+, Ce3+ and Gd3+)-Doped CaF2 [118]	NIR-II fluorescence imaging MRI photoacoustic imaging	
DCNP@DMSN-MoOx NPs [119]	NIR-II fluorescence imaging MRI CT	PTT chemodynamic therapy
PAA-NRs [120]	NIR-II fluorescence imaging CT	
NaYF4：Nd3+@NaLuF4 [52]	NIR-II fluorescence imaging CT	
LuPO4:Nd3+ [121]	NIR-II fluorescence imaging CT	
HANs [51]	NIR-II fluorescence imaging MRI photoacoustic imaging	PTT chemodynamic therapy

### 1.3 Establishing a multifunctional treatment platform for colorectal tumours with NIR-II fluorescence imaging

We know that multimodal NIR-II fluorescence imaging technology can resolve and localize lesions precisely. Based on this, imaging and treatment can be unified in one platform, integrating with vivo tracing, targeting and precision treatment to achieve combined diagnosis and treatment.

### 1.4 NIR-II fluorescence imaging guides photothermal therapy

**Fig. 5:**
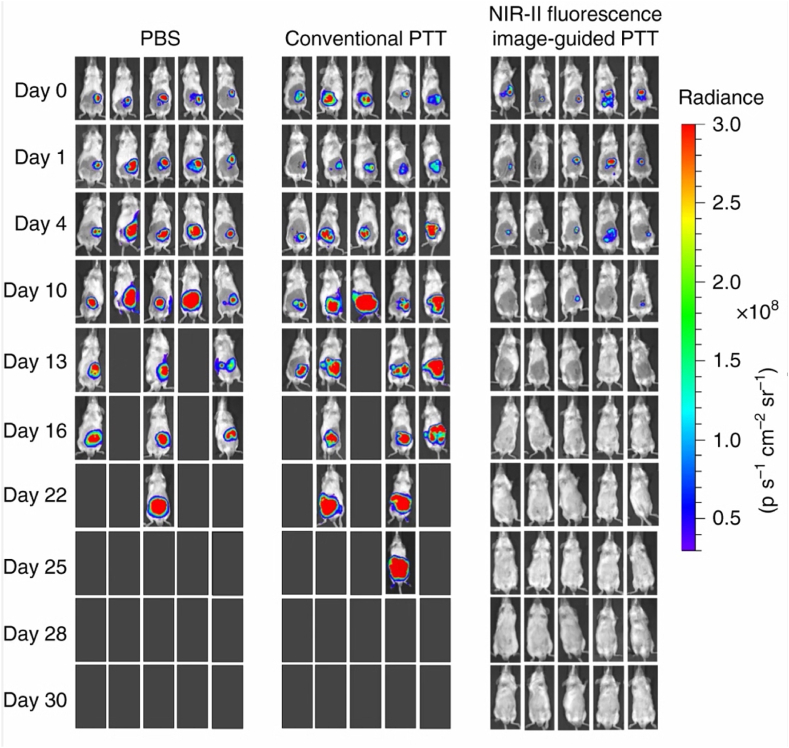
Vivo bioluminescence imaging of colon cancer mice before or after different treatments. In the PBS group, mice were injected with PBS only. In the conventional PTT group, mice were injected with BPBBT NPs and underwent PTT on the lesions observed with the naked eye only. In the NIR-II fluorescence image-guided group, mice were injected with BPBBT NPs and underwent PTT with NIR-II imaging. Finally, colon cancer mice that underwent NIR-II image-guided PTT were wholly cured within five days and without recurrence in 30 d.(Reproduced with permission from Gao, S. et al. [53]. Copyright 2019 Nature Communications.).

Photothermal therapy (PTT) is a disease treatment due to the photothermal action of Photothermal transduction agents (PTAs) that can convert light energy into thermal energy for focal cell destruction. Currently, NIR-II responsive biomaterials are widely developing for use in colorectal cancer treatment, such as organic conjugated polymers [53], metal-sulphur oxides [24], organic phase change materials (PCM) [54], gold nanomaterials [55] and carbon nanomaterials [56]. Gao, Wei et al. binding human serum albumin (HSA) to the Donor-Acceptor-Donor(D-A-D) based fluorophore probe BPBBT, which changes the planarity of the fluorophore and restricts its intramolecular rotation. The binding changes the equilibrium between the aggregation-induced emission(AIE) and the twisted intramolecular charge transfer(TICT) state of BPBBT, tailoring its fluorescence and photothermal efficiency. It depicts in situ mouse colorectal tumours and metastatic lesions as small as 0.5 mm × 0.3 mm in size, guided by NIR-II fluorescence images and provides optimized laser irradiation time, dose and area for photothermal ablation treatment [53], Fig. 5. Zheng, Wang et al. designed an injectable hydrogel with temperature sensitivity by dissolving photothermal materials and chemotherapeutic drugs into the hydrogel to combine local tumour photothermal and chemotherapy with reduced side effects on normal cells and tissues. The photothermal conversion efficiencies of NIR I and NIR II biological windows can reach 22.18 % and 31.42 %, respectively. Furthermore, the heat generated during the photothermal conversion can modulate the drug release rate, resulting in on-demand and sustained chemotherapy [57].

### 1.5 NIR-II fluorescence imaging guides photodynamic therapy

Photodynamic therapy (PDT) is also a safe, minimally invasive and highly effective photoactivated therapy with fewer side effects, high biosafety and high controllability [58]. The success of the PDT treatment mechanism is highly dependent on the concentration of photosensitizers (PS) in the tumour tissue and the specific targeting of subcellular accumulation. It can generate high enough levels of cytotoxic singly linear molecular oxygen and ROS effectively induce apoptosis or necrosis in tumour cell death, thereby destroying them. Researchers are working on developing a new generation of NIR-II light-activatable photosensitizers to obtain precise treatment of the colorectal. Kuthala, Shanmugam et al. enhanced the plasmonic field-field coupling and field-lattice coupling by adding saline solution, tuned the optical absorption from NIR-I to NIR-II, thereby strengthening the tumour-destroying effect of photodynamic therapy and prolonging the average survival time of colorectal cancer mice [59].

**Fig. 6:**
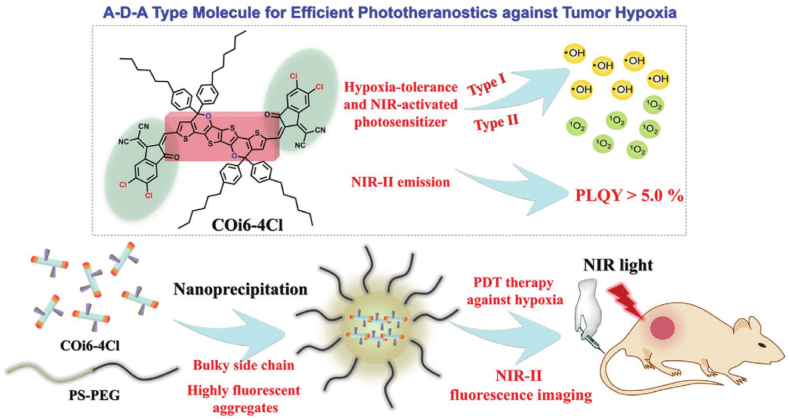
hypoxia-tolerant photodynamic theranostics of tumor. (Reproduced with permission from Li, L. et al., [60]. Copyright 2020 Advanced Materials.).

In addition, common photodynamic drugs have high oxygen-dependent properties, and the oxygen-depleted environment in tumour tissues is a fatal drawback of such photodynamic therapies. Li, Shao et al. developed a photodynamic drug with both type I and type II photodynamic processes under characteristic wavelength excitation, which achieve NIR-II imaging-guided tumour precision therapy under hypoxic conditions [60], Fig. 6.

Combining PDT with PTT is a synergistic treatment strategy to improve the efficacy of cancer treatment. The effect of using PTT can enhance the permeability of cell membranes, making it easier for photosensitizers to enter cancer cells. And the mild heat can also improve intra-tumoral circulation, increasing saturated oxygen concentration, which is also helpful for the production of singlet oxygen, thus enhancing the effect of PDT [61]. Combining PTT/PDT/imaging with drugs for optimal diagnosis/treatment is a promising technology.

### 1.6 NIR-II fluorescence imaging guides photoimmunotherapy

**Fig. 7:**
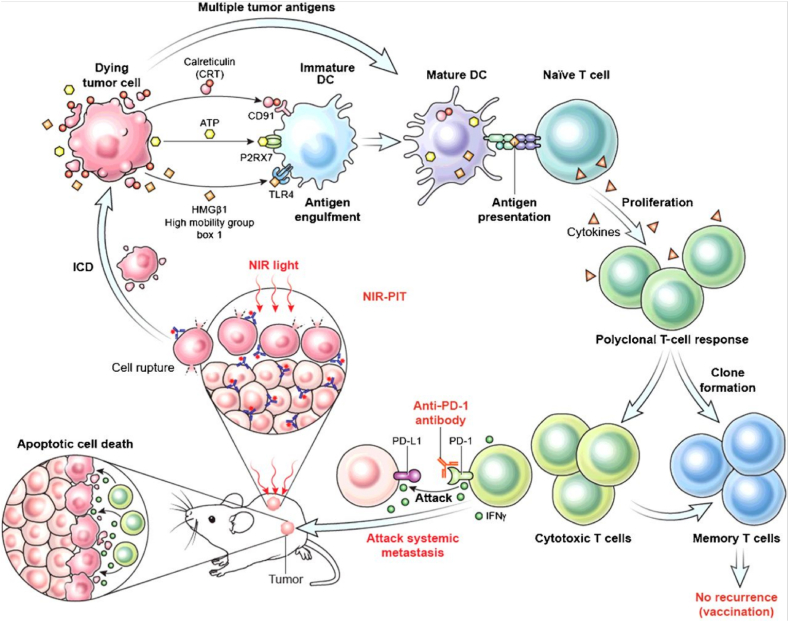
Proposed mechanism of combination therapy with NIR-PIT and systemic PD-1 mAb. (Reproduced with permission from Nagaya et al. [65]. Copyright 2020 Digestion.).

Near-infrared photoimmunotherapy (NIR-PIT) is a novel molecularly targeted therapy. Systemic injection of antibody-photo absorber conjugate (APC) combined with cognate receptors overexpressed on the surface of cancer cells activates photochemical reactions through NIR, resulting in lethal damage to cancer cells. And normal tissues are unaffected [[62], [63], [64], [65]], Fig. 7. At the same time, the contents released by cancer cells can facilitate the activation of the immune response and establish chronic immunity to destroy cancer cells [66,67]. A first-in-human phase I/II clinical trial of NIR-PIT targeting the epidermal growth factor receptor (EGFR) using cetuximab-IR700 (RM-1929) was completed [68].

Research demonstrates that targeted NIR-PIT can combine with conventional systemic immunotherapy, further activating CD8^+^ T cells and enhancing immune response [69]. Due to the rapid and massive killing of cancer cells near the tumour vasculature after NIR-PIT, resulting in a significant increase in vascular permeability immediately after treatment, the use of nanoscale anticancer drugs may be more effective than either treatment alone [[70], [71], [72], [73]]. Liu, Tian et al. combined NIR-II imaging with immunotherapy and photodynamic therapy to develop an organic nanoparticle with functional PD-L1 antibody for combined immune and photodynamic treatment of tumours on tumour-bearing mice, which were cured and recurrence-free after treatment [74]. In addition, circulating tumour cells (CTCs) are a mechanism of tumour metastasis. NIR-PIT may target CTCs with characteristic cell surface markers with specific APCs, which may be a future direction. There are few reports of NIR-II photoimmunotherapy for colorectal cancer, but its particular effect in tumour treatment will be a promising method for the new treatment of colorectal cancer.

### 1.7 NIR-II fluorescence imaging guides precisely colorectal cancer surgery

Precision surgery is a new paradigm of modern surgery based on scientific determinism. Precision surgery is a highly deterministic surgical practice based on a precise balance of three elements: lesion removal, organ protection and damage control to achieve the multi-objective optimization of safe, efficient and minimally invasive surgical treatment, and finally, to achieve the goal of maximum patient recovery.

Precision colorectal cancer surgery aims at maximum lesion clearance, maximum bowel protection, and minimal trauma clarity. Compared to conventional NIR-I, NIR-II fluorescence imaging shows superior resolution and tissue penetration, as well as a much lower background signal from ambient light and tissue autofluorescence. This is essential for precise surgical treatment. In colorectal cancer resection, NIR-II fluorescence imaging allows accurate assessment of anastomotic blood flow, lymph node tracing, visualization of tumour localization, protection of the ureter, and display of critical vessels. Currently, ICG's NIR-I imaging is widely used in clinical practice and is a safe, inexpensive and effective tool. However, extending its wavelength to the NIR-II can provide higher resolution and visualization of deeper tissues [75,76]. Hu, Fang et al. first guided human tumour resection with visible light, NIR-I and NIR-II fluorescence imaging. They found that intraoperative NIR-II imaging had a higher tumour detection rate and tumour detection sensitivity [77].

NIR fluorescence imaging has potential value in reducing the incidence of anastomotic leakage (AL) by altering the position of the "resection line" in radical colorectal cancer surgery and has proven to be safe and feasible [[78], [79], [80], [81], [82], [83], [84]]. According to a scoring system for perfusion assessment (Table 4 Perfusion Score Assessment) [85,86], the surgeon performs an intestinal blood flow assessment before and after the completion of the intestinal anastomosis to determine the intestinal "resection line" and to assess anastomotic perfusion. Salvador Morales et al. reported that NIR fluorescence imaging changed the colorectal "resection line" in 18.2 % of patients, reducing the incidence of AL after colorectal surgery to some extent [87]. A multicenter randomized controlled study found that ICG-NIR imaging effectively determines the blood supply to the colonic stump and anastomosis. Eleven per cent of patients in the trial group modified the surgical margins, but the difference in the incidence of AL between the two groups was not statistically significant [88]. It has preliminarily demonstrated the safety and reliability of assessing anastomotic blood flow in colorectal cancer surgery by ICG imaging techniques.

**Table 4:** Perfusion score assessment^a^^,^^b^.

Seroc	1	2	3	4	5
Fluorescence assessment^c^	No uptake	Patchy fluorescence	Significantly hypo-fluorescent but homogenous	Somewhat hypo-fluorescent compared to other segments	Iso-florescent to all other segments
Clinical assessment^d^	Dusky appearing	Patchy appearing	Pink appearing but no pulsatility or bleeding cut edges	Pink appearing, pulsatility of mesenteric vessels, and bleeding cut edges but clinical concern regarding viability	Pink appearing bowel, pulsatility of mesenteric vessels, bleeding from cut edge of bowel

a Sherwinter, D.A. et al. [86].

b If the score is ≥ 3, it means that the local blood flow of the anastomosis is adequate to more effectively avoid the occurrence of AL due to inadequate blood supply.

c A fluorescence score of 1–5 (1 indicating no uptake and 5 indicating maximal uptake).

d A clinical score of 1–5 (1 indicating a dusky appearance and 5 indicating pinkish pulsatility of the mesenteric vessels and active bleeding from the cut edge).

Lymph node metastasis is one of the essential metastatic routes of colorectal cancer. Intraoperative precise positioning of lymph nodes and guidance of the scope of dissection is beneficial to detect more lymph nodes, provide more accurate pTNM staging, and guide patients' postoperative treatment. For sentinel lymph nodes (SLN) in colorectal cancer, ICG can detect 88%–98 % with NIR-I imaging, but its false-negative rate is 18%–67 % [89].

Under multi-tissue real-time NIR-II fluorescence imaging guidance, the operator can remove the diseased tissue altogether while avoiding normal tissue damage. Under NIR-II, imaging clearly shows the critical surrounding anatomical structures (such as blood vessels and ureters). It effectively prevents medically induced injuries [90] and achieves more precise surgery. The occurrence of liver metastasis and peritoneal metastasis is one of the reasons for the poor prognosis of colorectal cancer. With the high resolution and penetration depth of NIR-II, it can effectively detect microscopic lesions, maximize the removal of metastases, and improve the long-term survival rate of patients. Yang et al. achieved intraoperative navigated lesion resection in a mouse tumour peritoneal metastasis model. The positive rate of 33 lesions resected by naked eye identification was only 62 %. In comparison, the positive rate of 44 lesions resected under NIR-II imaging guidance reached 84 %, indicating that NIR-II has a higher detection rate and accuracy in achieving the removal of microscopic peritoneal metastases [91]. Recently, Xiaoyong et al. constructed a mouse colorectal cancer subcutaneous tumour model (n = 15), an in situ model (n = 15), and a peritoneal metastasis model (n = 10) using a CEACAM5-targeted probe to identify CRCs and resected all of the tumours under NIR-II imaging guidance, even smaller than 2 mm tumours were detected [92].

## 2 Perspective and challenges

In recent years, with the continuous development and refinement of NIR fluorescence technology, its application in diagnosing and treating colorectal cancer has been expanded and deepened. Compared to the conventional NIR-I window (700–900 nm), NIR-II fluorescence imaging offers higher resolution and tissue penetration with a much lower background signal from ambient light or tissue autofluorescence. This is essential for precision diagnosis and treatment.

Several NIR-II fluorescent agents have been developed to meet clinical needs. However, most NIR-II fluorescent materials are still pre-clinical and far from the clinic. Furthermore, NIR-II fluorescent probes that illuminate tumour cells cannot rely solely on enhanced permeability and retention effects but require more effective tumour-targeting strategies to achieve more precise labelling at the molecular level. First, a higher signal-to-noise ratio is provided by increasing the signal intensity (by decreasing photon attenuation) and decreasing the biological background noise (by decreasing tissue scattering and autofluorescence), especially in the region beyond 1500 nm where photons exhibit very low tissue scattering and almost complete disappearance of tissue autofluorescence. Then, suitable elements are selected to reduce deposition in normal tissues, thereby reducing toxic effects. Furthermore, single-target molecular probes still have low signal-to-noise ratios and high false-positive rates due to the heterogeneity of tumours. Therefore, developing multi-target, more affinity molecular NIR-II reagents may be a feasible future research direction.

Various NIR-II luminescent dyes, including organic semiconductor fluorescent probes, inorganic semiconducting quantum dots, and rare earth ion-doped luminescent nanoparticles, have been successfully used for NIR-II multifunctional fluorescence imaging. However, in the direction of clinical translation, they may contain toxic heavy metals, have poor biocompatibility and slow excretion rates. It has led to a need for more commercially available or clinically approved NIR-II fluorophores with high brightness and biocompatibility. This situation is a significant bottleneck in adopting NIR-II fluorescence imaging methods in the clinical setting. However, the FDA-approved NIR-I dye ICG has demonstrated NIR-II emission spectroscopy. Therefore, contemporary research must focus on FDA-approved dyes to modify and develop new molecular probes, which accelerate the clinical translation process. Future NIR-II fluorescence imaging will effectively address the current problems in colorectal tumour imaging, effectively guide the precise resection of tumours, improve patient prognosis and reduce the medical burden on society and patients.

In biomedical research and clinical practice, any single detection tool has limitations. Combining multimodal NIR-II fluorescence imaging with other advanced diagnostic imaging methods is a powerful way to achieve a multidimensional and comprehensive approach to diagnosis and treatment. The development of these multimodal composite imaging techniques has facilitated structural localization and dynamic functional resolution of the colorectum. The different imaging modalities complement each other, making the imaging more and more complete and informative. Furthermore, with the trend towards minimally invasive surgery, relevant imaging systems must be integrated into laparoscopic or endoscopic devices to optimize tumour detection during colorectal surgery. However, integrating several imaging modalities into a single system is a great challenge due to differences in hardware and imaging mechanisms. Currently, no NIR–II–based imaging system combining more than two modalities has been reported, but such multimodal imaging systems hold great promise for obtaining complementary information. Although the field has been under development for over a decade, researchers still need to work in unison to bring it to the clinic.

The use of NIR-II in combination with multiple therapeutic modalities can effectively improve the treatment of colorectal cancer and enable patients to receive the most appropriate treatment and avoid unnecessary overtreatment to improve their quality of life. However, more prospective studies are needed to provide a clinical basis for its accuracy, completeness and safety in assessing the efficacy and establishing standardised protocols. We look forward to when NIR-II fluorescence imaging technology can significantly contribute to human healthcare.

## Data availability statement

No data was used for the research described in the article.

## Compliance with ethics guidelines

Yong Wu, Hong Cao, Shaoxun Yang, Chaohui Liu and Zhenguo Han declare no conflicts of interest. This manuscript is a review article and does not involve a research protocol requiring approval by the relevant institutional review board or ethics committee.

## Additional information

No additional information is available for this paper.

## CRediT authorship contribution statement

**Yong Wu:** Writing – original draft. **Hongtao Cao:** Investigation. **Shaoqing Yang:** Resources. **Chaohui Liu:** Resources. **Zhenguo Han:** Writing – review & editing.

## Declaration of competing interest

The authors declare that they have no known competing financial interests or personal relationships that could have appeared to influence the work reported in this paper.
